# Stabilization of Self-Pressurized Gelatin Capsules for Oral Delivery of Biologics

**DOI:** 10.3390/pharmaceutics17091156

**Published:** 2025-09-03

**Authors:** Amy J. Wood-Yang, Joshua I. Palacios, Abishek Sankaranarayanan, Mark R. Prausnitz

**Affiliations:** 1 School of Chemical and Biomolecular Engineering, Georgia Institute of Technology, Atlanta, GA 30332, USA; 2 Wallace Coulter Department of Biomedical Engineering at Georgia Tech and Emory University, Georgia Institute of Technology, Atlanta, GA 30332, USA

**Keywords:** oral delivery, biologic drug, effervescence, self-pressurized capsule, high-velocity aerosol, hard gelatin capsule, polymeric enteric coating

## Abstract

**Background/Objectives:** Oral delivery of biologics offers advantages for patient access and adherence compared to injection, but suffers from low bioavailability due to mucosal barriers and drug degradation in the gastrointestinal tract. We previously developed an oral self-pressurized aerosol (OSPRAE) capsule that uses effervescent excipients to generate CO_2_ gas, building internal pressure to eject powdered drug at high velocity across intestinal mucosa. **Methods:** Here, we developed two key design improvements: (i) an enteric covering to protect the capsule delivery orifice in gastric fluids and (ii) reduced humidity content of capsules to extend shelf-life. **Results:** Enteric-covered capsules prevented drug release in simulated gastric fluid and then enabled rapid release upon transfer to simulated intestinal fluid. Burst pressure for enteric-covered capsules was ~3–4 times higher than non-covered capsules. After storage for up to three days, the capsules’ effervescent excipients pre-reacted, making them unable to achieve high pressure during subsequent use. To address this limitation, we prepared capsules under reduced humidity conditions, which inhibited pre-reaction of effervescent excipients during storage, and a polyurethane coating to improve water uptake into the capsule to drive the effervescence reaction in intestinal fluid. **Conclusions:** These design improvements enable improved functionality of OSPRAE capsules for continued translation in pre-clinical and future clinical development.

## 1. Introduction

Biologics have become increasingly popular for treatment of chronic diseases, such as diabetes, autoimmune conditions, inflammatory diseases, and obesity [1,2,3,4]. However, biologics generally cannot be administered orally, making injection the standard route of administration. Oral delivery of biologics is impeded by the barriers of the gastrointestinal (GI) tract due to variable pH, enzymatic degradation, and inability to cross the intestinal mucus layer and epithelium. As a result, oral bioavailability of biologics is very low (e.g., <1%) [4,5]. However, orally administered biologics could significantly increase patient access to therapies, medication adherence, and quality of life, especially for those living with chronic diseases and requiring frequent injections. Examples of some commonly administered biologics that could benefit patients from oral delivery are etanercept, insulin, adalimumab, and pembrolizumab [6]. Moreover, injections using hypodermic needles create biohazardous sharps waste, require training to administer the injection, cause pain, and are constrained by needle-phobia [3]. Oral biologics delivery with higher bioavailability could overcome these limitations and increase the impact of biologic therapies [4].

Existing approaches to increase oral bioavailability for biologics utilize both chemical and physical methods. Chemical methods include formulating the drug with permeation enhancers that facilitate drug delivery across the intestinal mucosa, enzyme inhibitors, and functionalized nanoparticles, as well as chemically modifying the drug to resist enzymatic degradation [4,5]. Many of these chemical approaches come with safety concerns or do not sufficiently increase bioavailability [4,7]. For example, oral semaglutide formulated with the absorption enhancer sodium *N*-(8-[2-hydroxylbenzoyl] amino) caprylate (SNAC) achieved a bioavailability of just 1.2% in a dog model [8] and only 0.8% in human clinical trials [9].

Devices have also been engineered to use physical, rather than chemical, methods to increase bioavailability of oral biologics. Some of these technologies include microneedle- or micro-pillar-based injectors (SOMA [10], LUMI [11], RaniPill [12]) to mechanically bypass the mucus and epithelial cell barriers. Other devices have used robotic capsules (RoboCap [13]) which physically push away the mucus layer, or ultrasound to reversibly increase tissue permeability [7]. However, these devices have complex designs and manufacturing processes, since they involve the use of springs, motors, metal, and/or large, non-dissolvable parts. There could also be safety concerns with their use in the GI tract and subsequent release into environmental wastewater.

We previously developed a modified gelatin capsule that generates convective force to increase oral biologics bioavailability using an oral, self-pressurized aerosol (OSPRAE) [14]. This device uses a physical method of delivery related to jet injection, but accomplishes it using a capsule-based dosage form that can be manufactured by adapting conventional methods using water-soluble and biodegradable formulations with no moving parts and no mechanical or electrical components. We believe that the OSPRAE capsule novelty lies in its simplified fabrication, compared to similar devices.

The OSPRAE is a crosslinked hard gelatin capsule filled with drug and effervescent excipients—i.e., sodium bicarbonate (SB) and citric acid monohydrate (CA)–that produce CO_2_ gas to generate internal pressure in the capsule. The excipients only react when OSPRAE is placed in an aqueous environment, and the capsule only bursts at a pre-defined location (delivery orifice) to eject powdered drug from the capsule and across the intestinal mucosa. A biologic drug formulated as a powder can be ejected from the capsule at high velocity, which can be fine-tuned through controlling the degree of gelatin capsule crosslinking and changing the delivery orifice size. Unlike other devices that also use physical methods for oral delivery, OSPRAE is made out of conventional pharmaceutical excipients that can enable low-cost, large-scale manufacturing. Moreover, our previous in vivo study with exposed rat intestinal mucosa had comparable insulin pharmacokinetics and pharmacodynamics for OSPRAE delivery compared to subcutaneous injection, albeit with lower bioavailability [14].

The objective of this study was to improve the OSPRAE design to address two barriers that limit translation of the capsule to additional pre-clinical and future clinical studies. First, the original OSPRAE was not designed to resist passage through gastric fluids, which could lead to premature drug ejection in the stomach. Our first objective was therefore to add an enteric covering to the capsule delivery orifice, thereby delaying orifice failure and drug release until reaching the small intestine [15,16]. The second limitation of the original OSPRAE design is short shelf-life stability. Effervescent excipients are known to be reactive under elevated humidity and should be stored at low humidity to enable long-term stability. However, hard gelatin capsules cannot be stored at very low humidity, as the capsule shell becomes brittle [17,18]. The second objective was therefore to increase the shelf-life stability of OSPRAE, with the hypothesis that reducing water content inside the capsule (but not reducing it so far as to cause gelatin brittleness) can prevent excipient pre-reaction without adversely affecting the gelatin capsule shell. In this study, we address both objectives through modifying the OSPRAE capsule design and assessing outcomes through in vitro testing.

## 2. Materials and Methods

### 2.1. Materials

SB, CA, and pepsin from porcine gastric mucosa (pepsin activity of ≥250 units/mg) were purchased from Sigma Aldrich (St. Louis, MO, USA). Pancreatin powder (with amylase activity of ≥25 units/mg, lipase activity of ≥2 units/mg, and protease activity of ≥25 units/mg) was purchased from Spectrum Chemical (New Brunswick, NJ, USA). Methanol was obtained from JT Baker (Phillipsburg, NJ, USA). 1,4-dioxane (dioxane) and diethylene glycol dimethyl ether (diglyme) were obtained from Sigma Aldrich. Tecoflex EG85-A was a gift from Lubrizol Life Science Health (Wickliffe, OH, USA). Eudragit L100-55 was a gift from Evonik (Piscataway, NJ, USA). Hard gelatin capsules, size 000, were purchased from Capsuline (Dania Beach, FL, USA). RapidFix UV curable glue was purchased from Lighthouse for the Blind (St. Louis, MO, USA). Lacquer thinner was purchased from Klean Strip (Memphis, TN, USA). Clear Watco lacquer was purchased from Rust-Oleum (Vernon Hills, IL, USA). Simulated gastric fluid (SGF) without pepsin (0.7% *v/v* HCl, 0.2% *w/v* NaCl, pH 1.0–1.4) and simulated intestinal fluid (SIF) (0.5 M ionic strength, pH 6.8) were purchased from Ricca Chemical (Arlington, TX, USA). The exact SIF composition was not provided, but the manufacturer specified that it contains sodium hydroxide, water, and potassium dihydrogen phosphate and meets the requirements of the USP XXII standard. Pancreatin powder (amylase activity 25 USP U/mg, lipase activity 2 USP U/mg, protease activity 25 USP U/mg) was purchased from Spectrum Chemical (New Brunswick, NJ, USA).

### 2.2. Citric Acid Drying

CA was placed in microcentrifuge tubes in ~800 mg aliquots. The tubes were left open and placed in a vacuum oven at −762 mmHg and 55 °C for 3 nights. The tubes were transferred to a desiccator at room temperature (20–25 °C) for 1 week. The tubes were then closed and stored in the desiccator until use.

### 2.3. Capsule Manufacturing

The process of capsule manufacturing contained the following steps: crosslinking capsule body, coating capsule body and top with lacquer, forming a delivery orifice on the capsule body, coating the capsule body with Tecoflex (when indicated), coating the capsule body with Eudragit (when indicated), filling the capsule body, and adhering the capsule top.

#### 2.3.1. Crosslinking

The body and cap of hard gelatin capsules (size 000) were separated. The capsule bodies were placed on aluminum foil with double-sided tape (Scotch, 3M, St. Paul, MN, USA) and then crosslinked at 254 nm with a UV light (Sani Ray Model RRD-24-4S, Atlantic Ultraviolet Corporation, Hauppauge, NY, USA). During crosslinking, the capsules were placed 10 cm below the light for 50 h in a chamber at controlled humidity of 60–65% RH and room temperature. After crosslinking, a marker was used to indicate the side of the capsule that was facing upwards at the UV light. Further details on optimization of the crosslinking procedure and its relationship to stability in gastric fluids and tolerance of pressure can be found in our previous study [14].

#### 2.3.2. Lacquer Coating

After crosslinking, capsule tops and bodies were coated with clear lacquer (as described in our previous study [14]) and left to dry overnight in a controlled humidity chamber (60–65% RH, room temperature). After drying, the bottom of the capsule tops was trimmed with scissors. The capsule tops and bodies were inspected for defects, and any air bubbles or scratches were patched with a small dot of UV curable glue (RapidFix), cured using a 365/395 nm UV flashlight (ACISA Direct, Shenzhen, China) for ~10 s.

#### 2.3.3. Delivery Orifice Formation

Aluminum foil was placed on double-sided adhesive (3M), and a 0.5 mm hole was punched through both layers using a biopsy punch (Reusable rapid-punch biopsy kit, World Precision Instruments, Sarasota, FL, USA). This foil/adhesive mask was applied to the capsule body on the side previously exposed to UV light during crosslinking. The capsule body was supported on the cap end of a ballpoint pen during processing. Cotton-tip applicators (Puritan Medical Products, Guilford, ME, USA) were briefly pressed onto the adhesive (3M) to create a fuzzier texture, then dipped in lacquer thinner, then rubbed ~20 times over the 0.5 mm hole on the foil mask on the capsule. After peeling off the foil mask from the capsule, a non-fuzzy cotton-tipped applicator was used to wipe away residual lacquer from the delivery orifice. Delivery orifice diameters were measured to ensure they were within ±10% of 500 µm.

#### 2.3.4. Filling and Assembly

228 mg SB and 191 mg dried CA were mixed and then packed into the capsule body with a steel rod (9 mm). These amounts of SB and CA were chosen to allow for enough space in the capsule to insert the drug compartment, and were added in stoichiometric ratio, as described in our previous study [14]. Drug compartments were omitted from the capsule assembly for this study since they were not expected to affect capsule bursting behavior. UV curable glue (RapidFix) was applied on the inside of the capsule top, and the top was locked onto the body. The glue was cured for ~10 s with a UV flashlight. More UV curable glue was applied and cured with the flashlight around the outside of the capsule, where the top and body meet, to further seal the top to the body.

### 2.4. Tecoflex Coating

A solution containing 7.5% *w/w* Tecoflex EG-85A (polyether-based polyurethane, Lubrizol) was prepared in 9:1 *w/w* dioxane:diglyme and stirred overnight at 50 °C. To complete the dissolution, 1.9% *w/w* water was added and the solution was stirred for 2–3 h at 50 °C, then cooled to room temperature before use. Water was included to enhance surface wetting on the gelatin capsule, as inadequate wetting occurred without it. A concentration of 1.9% *w/w* was selected to minimize the risk of capsule dissolution.

A solution of 2.5% *w/w* Tecoflex was prepared by diluting 7.5% Tecoflex (1.5 mL from above) with 4.5 mL dioxane, adding water dropwise while stirring to reach 3.5% *w*/*w* water, then immediately adding 10% *w/w* ethanol. The final solution, which contained 2.2% *w*/*w* Tecoflex, was left to stir for 1 h before use.

Cotton-tipped applicators and tissue paper (Kimwipe, Kimberly-Clark, Irving, TX, USA) were dipped in lacquer thinner to selectively wipe away the lacquer coating from the bottom portion of the capsule body. Capsules were dip-coated six times in the 2.2% Tecoflex solution (≥10 min drying between coats), followed by three coatings of 7.5% Tecoflex solution (≥30 min drying time between coats). Capsules were left to dry overnight in a 60–65% RH chamber at room temperature to avoid low humidity exposure during manufacturing (which can lead to gelatin brittleness [19]).

### 2.5. Enteric Covering

After capsule bodies were coated in lacquer and/or Tecoflex and dried, and after the delivery orifice was formed, the capsule bodies were coated in a solution of either 7% *w/w* or 3.5% *w/w* Eudragit L100-55 (methacrylic acid-ethyl acrylate copolymer) (Evonik) in ethanol and dried at ambient conditions for up to 1 h. Capsule bodies were then coated with at least two more coats of Eudragit, with drying between coats. Capsules with 4.5 ± 0.2 mg, 13 ± 0.4 mg, or 26 ± 0.8 mg (*n* = 5) Eudragit per capsule were coated with three coats of 3.5% *w*/*w*, three coats of 7% *w/w*, or six coats of 7% *w/w* Eudragit in anhydrous ethanol, respectively. In this way, we formed an Eudragit covering over the delivery orifice.

### 2.6. Capsule Assembly in Dry Glove Box

A controlled humidity environment was maintained by using a two-port mini glove box (non-vacuum) with a humidity sensor and automatic purge control unit (Cleatech, Orange, CA, USA) connected to a nitrogen gas tank. Unassembled coated capsules containing the delivery orifice and excipients were placed in the glove box, and the nitrogen tank and humidity sensor were turned on to achieve 15–19% RH inside the glove box. The SB and CA were then mixed and loaded into capsules, as described above. The capsule bodies were sealed to the tops with UV curable glue and UV flashlight.

### 2.7. Capsule Storage

Assembled capsules were placed in heat-sealable foil pouches while in the glove box at 15–19% RH. Single-sided tape (Scotch, 3M) was used to temporarily seal the foil pouch. The foil pouches were then promptly removed from the glove box and heat-sealed using a tabletop impulse sealer (American International Electric, City of Industry, CA, USA). The pouches with capsules were stored at ambient conditions. Capsules prepared outside the glove box were similarly prepared in foil pouches and stored.

### 2.8. Water Uptake Determination

Capsules were assembled as described above, but with sodium citrate dihydrate and CA instead of SB and CA and were combined to simulate having similar excipients in the capsule, but without the effervescent reaction. A 25G hypodermic needle (5/8 inch, Dynarex, Montvale, NJ, USA) was then inserted into the top of the capsule. Capsules without excipients were similarly prepared. UV-curable glue was applied and cured with the UV flashlight to secure the capsule on the hypodermic needle. The capsule and connected hypodermic needle were weighed. The hypodermic needle was screwed onto an empty Luer lock syringe to allow it to be held in place in an in-house-made holder. The capsules were submerged in either SIF or SGF at 37 °C for 24 h. At predetermined time points, capsule/hypodermic needle assemblies were temporarily removed from the SIF or SGF and unscrewed from the syringe so the capsule/hypodermic needle mass could be measured.

### 2.9. Karl Fischer Water Content Measurement

Methanol was dried using molecular sieve (3A, 1–2 mm beads, Thermo Scientific, Waltham, MA, USA) to remove excess water for at least 3 days and then stored in a sealed bottle in a desiccator. Capsules were prepared as described above, then immediately cut open with a razor blade to separate the excipients from the gelatin. Water from excipients or gelatin was individually extracted in 10 mL methanol at 50 °C for 24 h while stirring in sealed glass vials. Samples of 100 µL were injected into a C20 Compact Karl Fischer Coulometer (Mettler Toledo, Columbus, OH, USA), and water content was determined.

### 2.10. In Vitro Testing in Simulated Gastric or Intestinal Fluid

Capsules were prepared with a pressure sensor and placed in SGF and/or SIF to determine the kinetics of pressure build-up and release. First, 500 mg pancreatin powder (Spectrum Chemical) was added to 50 mL SIF and placed on a stir plate at 37 °C, and 1600 mg pepsin powder (Sigma Aldrich) was added to 50 mL SGF and placed on the same stir plate.

Capsules were attached to a pressor sensor, as shown in Supplementary Figure S1. Before capsule assembly, a 25G hypodermic needle (0.5 mm × 25 mm, Becton Dickenson, Franklin Lakes, NJ, USA) was inserted in the lacquer-coated capsule top and removed. Next, a 16G hypodermic needle (1.6 mm × 25 mm, Becton Dickenson) was slowly inserted into the hole made by the 25G needle to make the hole in the capsule top bigger. Capsules were then assembled as described above using the capsule top with the hole. The quick-release connector of a PASPORT absolute pressure sensor PS-2107 (Pasco, Roseville, CA, USA) was adapted by gluing a 200 µL pipet tip to the end of the connector. The quick-release connector of the sensor was connected to the assembled capsule by inserting the pipet tip into the capsule and securing it with UV-curable glue. The total volume of the system with the connected pressure sensor was approximately 1.67 mL.

Pressure versus time was recorded using SPARKvue software (version 4.9.1, PASCO, Roseville, CA, USA) to take the baseline atmospheric pressure. Data collection was started, and then the capsule and attached connector were connected to the pressure sensor. The capsule was then fully submerged in either SGF or SIF. In some cases, the capsule was in SGF for 30 min or more and then immediately transferred to SIF. After data collection, the baseline atmospheric pressure was subtracted from the pressure readings of the capsule.

### 2.11. Data Normalization

Due to inconsistent humidity control in the lab, data for burst pressure and pressurization rate were normalized to account for variability associated with the different ambient humidity levels experienced at different times of year. We found that fresh (never stored) capsules prepared with the same materials and methods at different ambient humidity levels (e.g., in winter or summer) led to significantly different capsule performance in the laboratory (Supplementary Figure S2). When burst pressure or pressurization rate were measured for capsules after a period of storage time, the burst pressure or pressurization rate data (P_stored_) were normalized to data from freshly-made capsules made when the lab had the same average humidity as when the stored capsules were prepared on day 0 (P_fresh_) according to the following equation: P_normalized_ = P_stored_/P_fresh_.

## 3. Results

**Figure 1:**
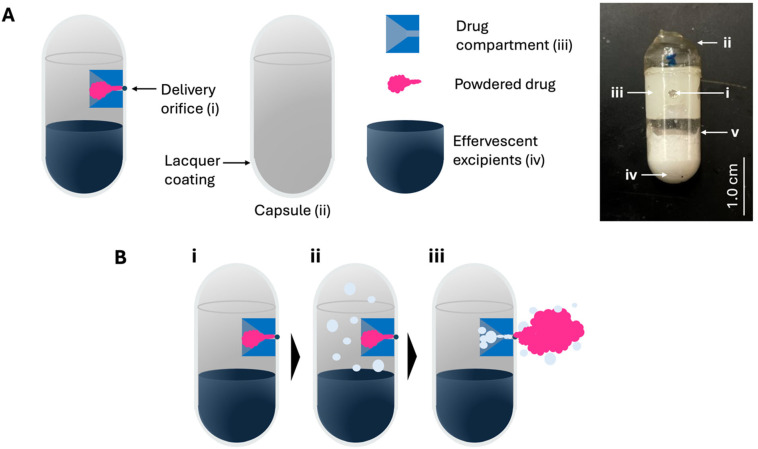
OSPRAE self-pressurization and drug delivery. (**A**) Components of OSPRAE design and corresponding image of an assembled capsule. Image shows delivery orifice (i), capsule top (ii), drug compartment (iii), effervescent excipients (iv), and a porous filter paper (v), which can be used to hold effervescent excipients in place. (**B**) Capsules (i) can pressurize when placed in aqueous media (e.g., SIF) by CO_2_ gas generation (ii), followed by pressure-induced failure of the delivery orifice and ejection of the powdered drug through the orifice (iii).

OSPRAE capsules were fabricated using UV-crosslinked gelatin capsules with a lacquer coating and containing a delivery orifice and effervescent excipients, and in some cases a drug compartment filled with powdered drug (Figure 1). The effervescent excipients (i.e., SB and CA) were loaded into the bottom of the capsule body, and the delivery orifice was created near the top as an engineered weak point in the capsule wall (Figure 1A). When included, the drug compartment was positioned adjacent to the delivery orifice and was filled with powdered drug. The capsule was designed to eject the drug out of the capsule upon failure of the delivery orifice due to elevated pressure in the capsule. This pressure elevation is caused by effervescent gas generation upon entry of water from surrounding intestinal fluid (Figure 1B). For simplicity, drug compartments were not included in the capsules tested for the remainder of this study, since the drug compartment was not expected to affect capsule bursting behavior.

### 3.1. Enteric Covering to Prevent OSPRAE Delivery in the Stomach

OSPRAE is intended to deliver the drug in the intestine, where the capsule is in close contact with the intestinal mucosa, such that high-velocity drug ejection can penetrate the mucosal barriers. This approach is unlikely to work well in the stomach, where the OSPRAE is not necessarily near the stomach wall, where effective drug ejection would need to occur. We therefore sought to assess and modify OSPRAE design to prevent premature drug ejection in the stomach.

**Figure 2:**
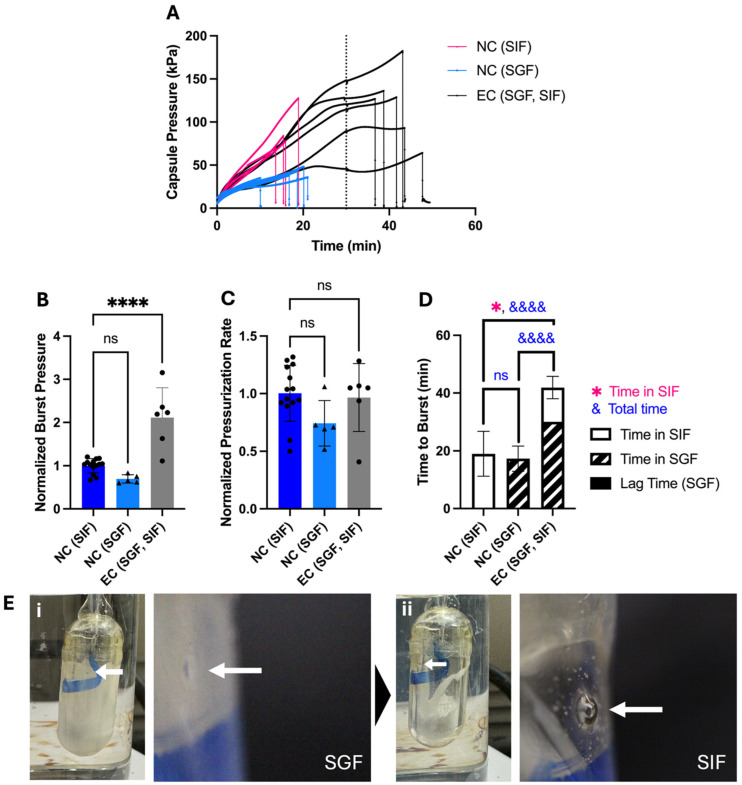
The effect of enteric-covered delivery orifice on bursting behavior of OSPRAE capsules in SGF and/or SIF. (**A**) Capsule pressure is shown as a function of time for non-covered capsules placed in SIF (NC (SIF)) or SGF (NC (SGF)) or enteric-covered capsules placed in SGF for 30 min and then immediately transferred to SIF (EC (SGF, SIF)). The dotted line indicates 30 min, when the EC capsules were transferred from SGF to SIF. (**B**–**D**) Capsule burst pressure, pressurization rate, and time to burst were calculated from the data in part (**A**). Data were normalized to NC capsules in SIF. One-way ANOVA was used to determine significance (ns: *p* > 0.05, &&&& or **** *p* < 0.0001, *n* = 5–14 replicates per group). Welch’s *t*-test was also used in part (**D**) to determine significance (* *p* < 0.05, *n* = 6–14 replicates per group). Statistical significance is shown for comparisons of time to burst just while in SIF (*) and total time until burst (&). (**E**) Representative images (shown at low and high magnification) of EC capsules in SGF (i) and after transferring from SGF to SIF (ii). Arrows indicate the delivery orifice. Capsules contained dried CA and SB, and were coated with 13 mg enteric covering per capsule.

When placed in either SGF or SIF, OSPRAE pressure increased over the course of about 10–20 min and then burst (Figure 2A). OSPRAE burst time in SGF was 17.3 ± 4.39 min and in SIF was 19.0 ± 7.75 min (Figure 2D). OSPRAE burst time was not significantly different in SGF versus SIF (one-way ANOVA, *p* > 0.05) because water can enter the capsule when in either fluid, and the effervescent excipients can start reacting as soon as they get wet. The burst pressure (Figure 2B) and pressurization rate (Figure 2C) of OSPRAE capsules in SGF and SIF were also not significantly different (one-way ANOVA, *p* > 0.05).

Prior studies suggest that OSPRAE pressurization in SGF could differ from SIF due to the lower pH in SGF that can drive the SB effervescence reaction [4,20,21]. However, we did not see evidence of this in the OSPRAE pressurization study above (Figure 2). This finding is further confirmed by a control experiment with OSPRAE capsules containing only SB without CA and incubated in SGF (i.e., at pH 1.0–1.4, serving as the only source of acid for the effervescence reaction). In this experiment, we did not see evidence of any pressure increase, indicating that SGF was not able to drive the SB effervescence reaction in our system (Supplementary Figure S3).

To prevent OSPRAE burst in the stomach, we could prevent water from entering the capsule from SGF, or we could prevent the capsule from bursting when in SGF. Since preventing water uptake would be difficult, we elected to selectively coat the capsule with a material to strengthen the delivery orifice in SGF, but would disappear in SIF, so that the capsule could burst in the intestine. We accomplished this by using an enteric covering on the delivery orifice from the Eudragit family of methacrylic acid co-polymers, which are commonly used on hard gelatin capsules [15,16]. Eudragit L100-55 was chosen since it dissolves above pH 5.5, allowing for drug release in the upper to mid small intestine [16,22].

We found that enteric-covered OSPRAE did not burst while in SGF for 30 min (Figure 2A), consistent with our hypothesis. After 30 min in SGF, capsules were immediately transferred to SIF to simulate passage from the stomach to the small intestine. Upon transfer to SIF, OSPRAE burst in 11.9 ± 3.89 min (Figure 2D), supporting the hypothesis that enteric-covered OSPRAE could achieve selective and rapid drug delivery in the small intestine after passage through the stomach.

Burst time of enteric-covered OSPRAE in SIF after pre-incubation in SGF was significantly faster than the burst time for non-covered capsules placed in SIF without pre-incubation in SGF (19.0 ± 7.75 min, Welch’s *t*-test, *p* = 0.0146). This indicates pre-incubation in SIF expedited OSPRAE failure, probably due to already-elevated pressure in the capsule (due to pre-incubation in SGF) upon placement in SIF.

The OSPRAE pressurization rate (until it plateaued) was similar for enteric-covered capsules compared to non-covered capsules (Figure 2C), which suggests that the enteric covering did not affect the uptake of water from SGF, since water uptake kinetics should largely control pressure increase kinetics. OSPRAE burst pressure was approximately 2–3 times higher for enteric-covered capsules compared to non-covered capsules (Figure 2B). This was likely because enteric-covered capsules had pressure build-up for 30 min in SGF before additional pressure build-up in SIF until they burst, allowing effervescent excipients to react for a longer time and achieve a higher pressure before burst.

Further understanding of how enteric covering prevents premature burst in the stomach comes from images of the OSPRAE delivery orifice in SGF and SIF (Figure 2E). In SGF, the enteric covering is undissolved and covers the delivery orifice, while in SIF, the enteric covering dissolves and leaves the delivery orifice exposed. When in SGF, we found that the delivery orifice did not deflect outwards due to the mechanical strength of the enteric covering (Figure 2E(i)). In contrast, when in SIF (where the enteric covering dissolved off), the orifice bulged outward (Figure 2E(ii)), eventually leading to its burst.

### 3.2. Effect of Enteric Covering Thickness and Stomach Incubation Time on OSPRAE Delivery

**Figure 3:**
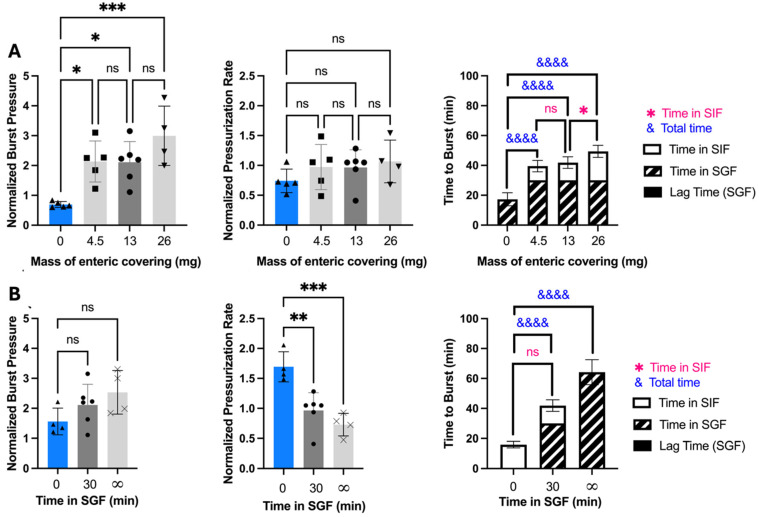
The effect of enteric covering thickness and SGF incubation time on bursting behavior of OSPRAE capsules. Capsule burst pressure, pressurization rate, and time to burst are shown as a function of (**A**) mass of enteric covering per capsule after being incubated in SGF for 30 min and then transferred to SIF or (**B**) incubation time in SGF before transfer to SIF: 0 min (SIF only), 30 min (SGF then SIF), or infinite time (only SGF). In part (**B**), capsules that burst at locations other than the delivery orifice are represented by X symbol data points. Data were normalized to non-covered capsules. Capsules contained CA and SB, and were coated with 13 mg enteric covering per capsule in part (**B**). One-way ANOVA in parts (**A**,**B**) or Welch’s *t*-test in part (**B**) (ns: *p* > 0.05, * *p* < 0.05, ** *p* < 0.01, *** *p* < 0.001, &&&& *p* < 0.0001) were used to determine significance (*n* = 4–6 replicates per group). Statistical significance is shown for comparisons of time to burst just while in SIF (*) and total time until burst (&).

We next determined the effect of varying the amount of enteric covering on burst pressure to determine if capsules would still delay release while in the stomach. Capsules with different amounts of enteric covering were incubated in SGF for 30 min and then transferred to SIF (Figure 3A). Burst pressure was significantly higher for capsules with enteric covering when compared to capsules without (one-way ANOVA, *p* < 0.05), but there was no significant difference in burst pressure among the capsules with different amounts of enteric covering (one-way ANOVA, *p* > 0.05).

Pressurization rate was also not significantly different among enteric-covered capsules (one-way ANOVA, *p* > 0.05), and was also not significantly different from non-covered capsules (one-way ANOVA, *p* > 0.05) (Figure 3A). To support the expectation that pressurization rate is related to water absorption rate, we directly measured water uptake by capsules with and without enteric covering and found that there was no significant difference (Welch’s *t*-test, *p* > 0.05) (Supplementary Figure S4A). This indicates that absorption of water into the capsule was not affected by enteric covering.

Time to burst after transfer from SGF to SIF depended on the amount of enteric covering (Figure 3A). Capsules with enteric covering had significantly longer time to burst compared to those without coating (one-way ANOVA, *p* < 0.0001), and time to burst increased with increasing mass of enteric covering (one-way ANOVA, *p* < 0.01). The time to burst is controlled by the pressurization rate as well as the dissolution rate of the enteric covering, since the delivery orifice cannot burst until the enteric covering dissolves in SIF. Since the pressurization rate did not increase with the amount of enteric covering, we believe that the longer time to burst associated with greater amounts of enteric covering was therefore caused by the increased time it takes to dissolve thicker enteric coverings, which delayed delivery orifice failure.

We were also interested in assessing the effect of incubation time in SGF to determine how gastric emptying time might affect OSPRAE burst behavior. We kept enteric-covered capsules in SGF for 0 min. (SIF only), 30 min (and then transferred to SIF), or infinite time (SGF only, until burst). The results demonstrated that burst pressure trended toward increased values (but without statistical significance, one-way ANOVA, *p* > 0.05, pressurization rate significantly decreased (one-way ANOVA, *p* < 0.001), and time to burst significantly increased with incubation in SGF (one-way ANOVA, *p* < 0.0001) (Figure 3B). This indicates that gastric emptying time could affect OSPRAE behavior.

Additionally, the time to burst for enteric-covered capsules upon transfer to SIF after 30-min SGF incubation was not significantly different from the time to burst for enteric-covered capsules placed in SIF without pre-incubation in SGF (Welch’s *t*-test, *p* > 0.05) (Figure 3B). This indicates that the dissolution time of enteric covering in SIF was not affected by pre-incubation in SGF. This indicates that the mass of enteric covering could be used to control the time to burst in the small intestine, even with variability in gastric emptying time.

**Figure 4:**
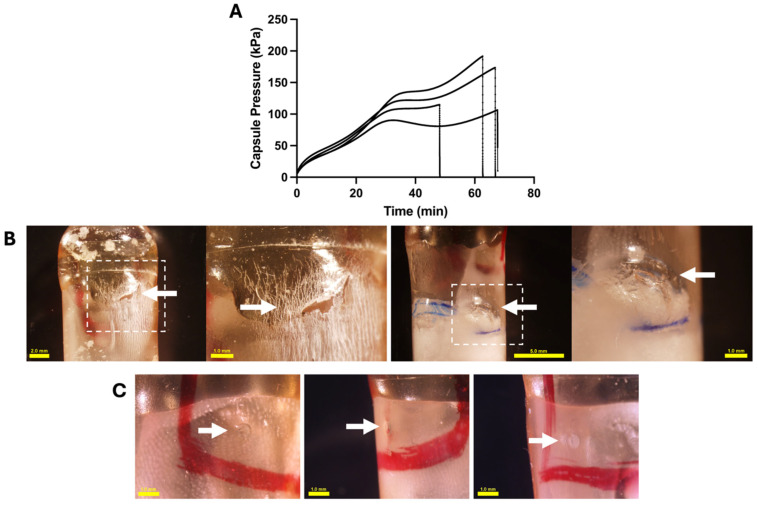
Bursting behavior of enteric-covered OSPRAE capsules incubated in SGF indefinitely. (**A**) Capsule pressure is shown as a function of time for four capsules containing an embedded pressure sensor using data from Figure 4B. Representative images of capsules without an embedded pressure sensor after they burst during incubation in SGF, showing (**B**) failure locations on the capsule body (indicated by arrows) at two different magnification levels and (**C**) the delivery orifice (indicated by arrows), which appears to be intact. Capsules contained CA and SB, and were coated with 13 mg enteric covering per capsule.

These data can be better understood by closer examination of the capsules incubated in SGF indefinitely. We found that the pressurization rate decreased with increasing time in SGF (Figure 3B), which can be explained by examining the dependence of capsule pressure over time, where the pressurization rate was initially faster and then slowed down at later times (Figure 4A). In addition, the capsules incubated in SGF for infinite time did not burst at the orifice, and the enteric covering did not dissolve, which is consistent with OSPRAE design. Instead, these capsules SGF failed at the connection between the pressure sensor and the capsule, which is an artifact of our measurement apparatus. Without that artifact, the capsule burst time in SGF would likely be longer.

To assess OSPRAE pressurization behavior without the artifact caused by the pressure sensor connection, we incubated capsules in SGF indefinitely without a pressure sensor inserted and found that they failed after 61.4 ± 9.08 min (*n* = 3 replicates). The failure spot of these capsules was found in the body of the capsule (Figure 4B) and not at the delivery orifice, which remained intact and covered by enteric covering (Figure 4C). This suggests that non-uniformities in capsule body properties may provide points of weakness that can fail at very high pressures.

### 3.3. Shelf-Life Stability of OSPRAE Capsules

A second barrier that prevents translation of the OSPRAE capsule is its short shelf-life stability. Previous studies with OSPRAE used capsules immediately after fabrication, but capsules require at least short-term storage to facilitate pre-clinical and clinical studies, and long-term storage stability for regulatory approval and clinical use. We chose stability for three days as an initial benchmark for establishing shelf-life stability to facilitate ongoing studies.

**Figure 5:**
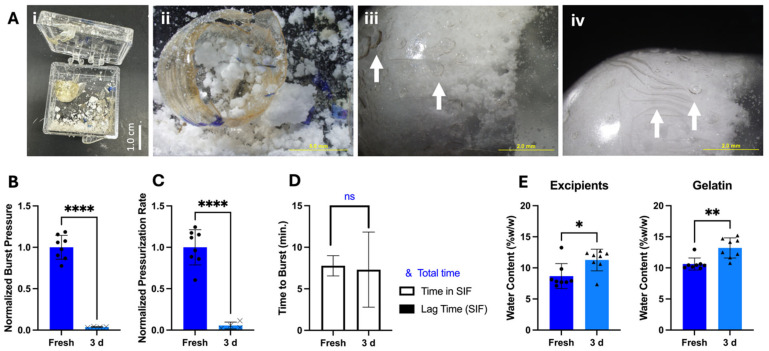
The effect of storage on bursting behavior of non-covered (NC) OSPRAE capsules in SIF. (**A**) Representative images of capsules after storage. (i,ii) Capsule remnants showing shattered gelatin and clumpy excipients in capsules that exploded during storage. (iii,iv) Capsule walls showing cracks and holes on the surface after storage (indicated by arrows). Images are representative of at least 8 replicates. (**B**) Burst pressure, (**C**) pressurization rate, and (**D**) time to burst for fresh and stored capsules. In (**B**–**D**), capsules after storage were tested in vitro in SIF. Data were normalized to NC capsules in SIF. In part (**B**,**C**), capsules that burst at locations other than the delivery orifice are represented by X symbol data points. (**E**) Water content of the capsule excipients or gelatin capsule was measured before and after storage. Welch’s *t*-test determined statistical significance (ns: *p* > 0.05, * *p* < 0.05, ** *p* < 0.01, **** *p* < 0.0001, *n* = 4–8 replicates per group). Capsules contained non-dried CA and SB, but no enteric covering, and were stored for 3 days at room temperature, pressure, and humidity.

We found that storage of OSPRAE capsules with our original design for three days at room temperature and humidity led to poor outcomes. Some of the capsules appeared to have undergone pre-reaction of effervescence, likely due to exposure to humidity, which built up so much internal pressure that the capsules exploded and broke the box in which they were contained (Figure 5A(i)). The residual SB and CA appeared clumpy and moist (Figure 5A(ii)), unlike before storage.

The remaining capsules that had not exploded had cracks and holes in the surface, specifically at the parts of the capsule that were touching the excipients (Figure 5A(iii–iv)). When these capsules were incubated in SIF, they burst at locations other than the delivery orifice, such as at the cracks/holes in the capsule in the capsule wall. They had significantly lower burst pressure compared to fresh capsules (Welch’s *t*-test, *p* < 0.0001) (Figure 5B). The pressurization rate was also significantly lower after storage (Welch’s *t*-test, *p* < 0.0001) (Figure 5C), further suggesting that the excipients had already reacted during storage. Time to burst (Figure 5D) was similar before and after storage (Welch’s *t*-test, *p* = 0.8516), and there was no lag time in pressure build-up upon placement in SIF.

Further analysis of these capsules by Karl Fischer measurement (Figure 5E) showed that the excipients used to manufacture capsules started with 8.68 ± 2.0% *w/w* water content, and the empty gelatin capsules started with 10.62 ± 0.97% *w/w* water content. After 3-day storage, excipient water content increased by 2.59 ± 0.27% *w/w,* and for the gelatin capsule walls, water content increased by 2.58 ± 0.65% *w/w* (*n* = 8). Because the autocatalytic reaction between SB and CA is initiated by water and generates more water [23], water generation by the effervescence reaction could have caused the gelatin and excipients to gain water.

### 3.4. Increasing Shelf-Life Stability by Pre-Drying Citric Acid

We hypothesized that reducing capsule water content during storage could prevent effervescence pre-reaction. However, water content reduction had to be balanced to avoid levels low enough to cause gelatin brittleness [19]. We decided to reduce water content during storage by two approaches: (1) pre-drying the CA by vacuum drying at elevated temperature and then storing with desiccant before assembly into the capsules (unlike in the original OSPRAE design [14] in Figure 5), and (2) assembling capsules in a controlled-humidity glove box (RH = 15–19%). CA is known to be significantly more hygroscopic than SB [23], so removing the adsorbed water in CA should be more effective in reducing excipient reactivity.

**Figure 6:**
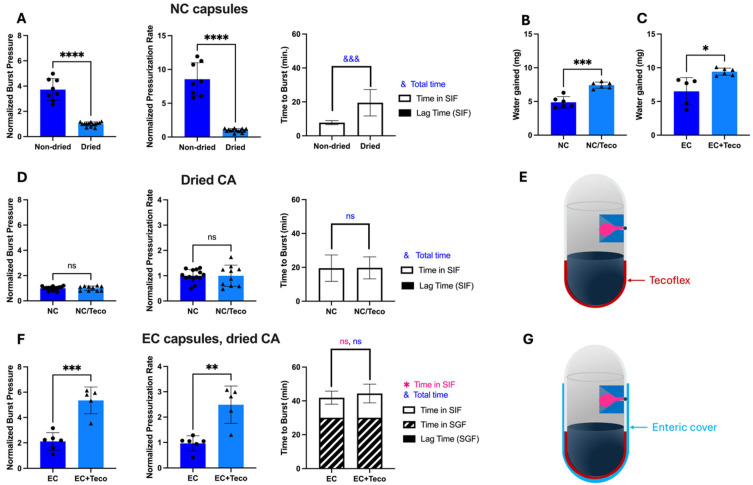
The effect of using dried CA and of adding Tecoflex coating on OSPRAE capsule performance. (**A**) Capsules without enteric covering (NC) containing either dried or non-dried CA with SB were tested in SIF. Data were normalized to NC capsules containing dried CA and SB in SIF. (**B**) Water uptake was measured for NC capsules or NC/Teco capsules in SIF after 1 h. Capsules contained model excipients (CA and sodium citrate dihydrate). (**C**) Water uptake was measured for enteric-covered (EC) capsules or EC/Teco capsules in SGF after 1 h. Capsules contained model excipients (CA and sodium citrate dihydrate) and 13 mg enteric covering per capsule. (**D**) NC or NC/Teco capsules containing dried CA and SB were tested in SIF. Data for NC were normalized to NC capsules containing dried CA and SB in SIF. Data for NC/Teco were normalized to NC/Teco capsules containing dried CA and SB in SIF. (**E**) The diagram shows the location of Tecoflex coating on NC capsules. (**F**) EC or EC/Teco capsules containing dried CA and SB were tested by incubating in SGF for 30 min, then transferring to SIF. Capsules contained 13 mg enteric covering. Data for EC were normalized to NC capsules containing dried CA and SB. Data for EC/Teco were normalized to NC/Teco capsules containing dried CA and SB. (**G**) The diagram shows the location of Tecoflex and enteric covering for EC/Teco capsules. Welch’s *t*-test determined statistical significance (ns: *p* > 0.05, * *p* < 0.05, ** *p* < 0.01, &&& or *** *p* < 0.001, **** *p* < 0.0001, *n* = 5–14 replicates per group).

Using this approach, CA was pre-dried, resulting in a loss of 7.50 ± 0.71% *w*/*w* water (*n* = 6). After mixing the dried CA with SB (having a water content of 3.32 ± 0.44% *w/w* water (*n* = 4), fresh capsules tested in vitro for bursting behavior in SIF (Figure 6A) showed significantly lower burst pressure (Welch’s *t*-test, *p* < 0.0001), lower pressurization rate (Welch’s *t*-test, *p* < 0.0001), longer time to burst (Welch’s *t*-test, *p* = 0.0001), and no lag time before bursting.

These data indicate that the reactivity of CA upon drying was significantly reduced. Low excipient reactivity is desirable for extending shelf-life storage, but is less desirable for drug delivery, when high burst pressure is needed to penetrate mucosal barriers. The normalized burst pressure with dried CA in Figure 6A corresponds to an absolute burst pressure of only 44.9 ± 21 kPa, which is 62% lower than capsules with non-dried CA (118.5 ± 45 kPa). The burst pressure with dried CA would not be high enough to penetrate intestinal mucosa [14].

### 3.5. Increasing Shelf-Life Stability by Using Tecoflex Coating

It appears that the bursting behavior was influenced by both the amount of residual water in the capsules as well as the water that enters capsules from SIF. Since we reduced the water content in the capsules (to provide stability during storage), we decided to increase the rate of water entry into capsules from SIF (to more rapidly increase water content for drug delivery). We identified Tecoflex as an alternate hydrophobic coating [24,25,26] instead of the lacquer used in the original OSPRAE design that would allow more water uptake across the capsule wall, but still remain rigid and prevent dissolution of the gelatin. Tecoflex is an aliphatic polyether-based thermoplastic polyurethane that is used in many invasive medical products [27], is expected to be strong due to its value on the Shore durometer hardness scale [28], and is expected to have greater water permeability because of the presence of many soft copolymer segments [29,30].

As expected, replacing lacquer with Tecoflex enabled capsules to gain more water when placed in SIF or SGF (Figure 6B,C, Supplementary Figure S5A(iii,iv),B(iii,iv)). The Tecoflex coating was only applied to the bottom of the capsule body, adjacent to the excipients, and lacquer was applied to the rest of the capsule body and top. Tecoflex-based capsules with dried CA did not show improvement compared to lacquer-based capsulate, exhibiting similar burst pressure (Welch’s *t*-test, *p* > 0.05) and pressurization rate (Welch’s *t*-test, *p* > 0.05) (Figure 6D,E). However, when evaluating enteric-covered capsules with dried CA (which is our main interest for drug delivery), Tecoflex allowed for significantly higher burst pressure (Welch’s *t*-test, *p* < 0.001) and pressurization rate (Welch’s *t*-test, *p* < 0.01) than without Tecoflex, so this design was further pursued (Figure 6F,G). The final composition (*w/w*) of the empty capsule shell (body and cap) for the design shown in Figure 6G was 75% gelatin, 16% lacquer, 2% Tecoflex, and 7% enteric coating.

To better understand water uptake by OSPRAE capsules, we studied the effect of water transport by osmosis in addition to diffusion (Supplementary Figure S5). By preparing OSPRAE capsules containing no CA or SB excipients, we removed the driving force for water uptake by osmosis due to the high (i.e., saturated) concentration of excipients that would be present inside the capsule. We found that water uptake in capsules containing excipients was significantly greater than in capsules without excipients when using capsules with Tecoflex but not when using capsules without Tecoflex. In this study, we used dried CA and sodium citrate dihydrate as non-effervescent excipients (at the same concentration as the dried CA and SB used in our usual OSPRAE capsules) to provide an osmotic driving force without the added effects of effervescence. Altogether, these data indicate that the osmotic driving force plays a significant role in water uptake in capsules with Tecoflex. This finding could explain the higher burst pressure and pressurization rate for these capsules seen in Figure 6F.

**Figure 7:**
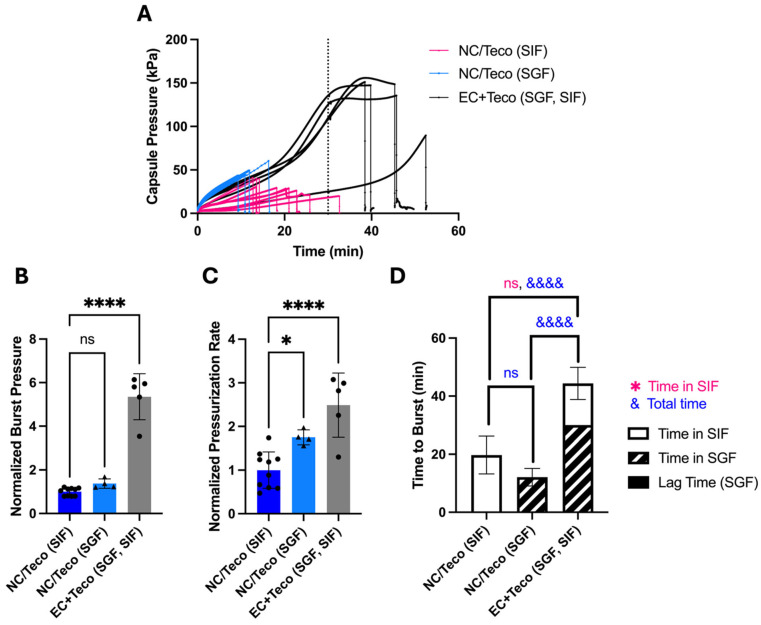
The effect of enteric covering on bursting behavior of OSPRAE capsules with Tecoflex in SGF and/or SIF. (**A**) Capsule pressure is shown as a function of time for non-covered capsules placed in SIF (NC/Teco (SIF)) or SGF (NC/Teco (SGF)) or enteric-covered capsules placed in SGF for 30 min and then immediately transferred to SIF (EC/Teco (SGF, SIF)). The dotted line indicates 30 min, when the EC/Teco capsules were transferred from SGF to SIF. (**B**–**D**) Capsule burst pressure, pressurization rate, and time to burst were calculated from the data in part (**A**). Data were normalized to NC/Teco capsules in SIF. Statistical significance is shown for comparisons of time to burst just while in SIF (*) and total time until burst (&). One-way ANOVA was used to determine significance (ns: *p* > 0.05, * *p* < 0.05, &&&& or **** *p* < 0.0001, *n* = 4–10 replicates per group). Welch’s *t*-test in part (**D**) was also used to determine significance (ns: *p* > 0.05, *n* = 5–9 replicates per group). Capsules contained dried CA and SB and 13 mg enteric covering per capsule (for EC and EC/Teco).

Based on positive results with dried CA in capsules with Tecoflex, we further examined their behavior in SIF and SGF. Similar to regular OSPRAE capsules, the capsules with Tecoflex needed enteric covering to delay release while in SGF (Figure 7A). Burst pressure (Figure 7B) and pressurization rate (Figure 7C) were also significantly higher with enteric-covered capsules than non-enteric-covered capsules with Tecoflex, likely because both Tecoflex (Supplementary Figure S5) and enteric covering (Supplementary Figure S4B) allowed capsules to absorb more water.

**Figure 8:**
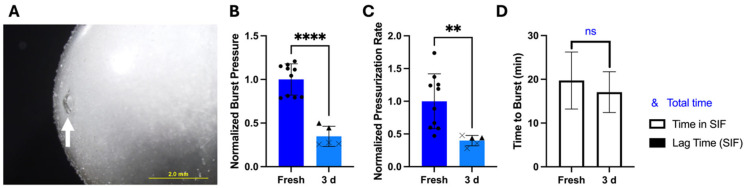
The effect of storage for 3 days on bursting behavior of non-covered OSPRAE capsules containing Tecoflex (NC/Teco) in SIF. (**A**) Representative image of a hole in a gelatin capsule wall after storage, indicated by white arrow. (**B**–**D**) Capsules after storage were tested in SIF. Data were normalized to NC/Teco capsules in SIF. In parts (**B**,**C**), capsules that burst at locations other than the delivery orifice are represented by X symbol data points. Welch’s *t*-test determined statistical significance (ns: *p* > 0.05, ** *p* < 0.01, **** *p* < 0.0001, *n* = 5–10 replicates per group). Capsules contained dried CA and SB, but no enteric covering, and were stored at room temperature, pressure, and humidity.

By reducing water content in dried CA and maintaining high-pressure burst behavior by using a Tecoflex coating, we hypothesized that these modified OSPRAE capsules would be able to retain drug delivery performance even after storage. After storage for 3 days, none of the modified capsules exploded during storage or had visible cracks in the gelatin. However, capsules still had small holes visible in the gelatin (Figure 8A), and 60% (3/5) of capsules burst at these holes instead of the delivery orifice when tested in SIF. Burst pressure (Welch’s *t*-test, *p* < 0.0001; Figure 8B) and pressurization rate (Welch’s *t*-test, *p* < 0.01; Figure 8C) were also significantly lower than fresh capsules, implying that the excipients had already reacted during storage. There was no lag time in pressure increase after storage, and time to burst was not significantly different between fresh and stored capsules (Welch’s *t*-test, *p* > 0.05, Figure 8D).

### 3.6. Increasing Shelf-Life Stability by Fabrication and Storage at Reduced Humidity

Since pre-drying CA was insufficient to prevent premature excipient pre-reaction during storage, we hypothesized that the water content of the air trapped inside the capsule should be reduced. Capsules with Tecoflex and dried CA were therefore prepared in a controlled-humidity glove box (15–19%RH) and then stored at 15–19%RH to reduce the amount of water that could be absorbed by the CA from the air. We did not reduce humidity further because gelatin capsules become brittle at lower humidity levels [18,19,31].

**Figure 9:**
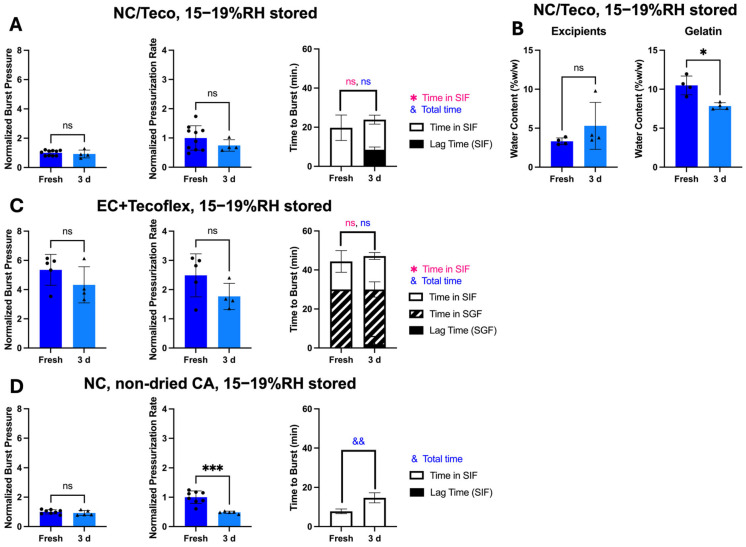
The effect of storage for 3 days at reduced humidity on bursting behavior of OSPRAE capsules in SIF. (**A**) Non-covered OSPRAE capsules containing Tecoflex (NC/Teco) were tested in SIF after storage. Capsules contained dried CA and SB, but no enteric covering. Data were normalized to NC/Teco capsules in SIF. (**B**) Water content was measured in excipients and gelatin capsule walls from NC/Teco capsules by Karl Fischer titration. (**C**) Enteric-covered OSPRAE capsules containing Tecoflex (EC/Teco) were tested after storage by incubating in SGF for 30 min and then transferring to SIF. Capsules contained dried CA and SB, and 13 mg enteric covering per capsule. Data were normalized to NC/Teco capsules in SIF. (**D**) Non-covered OSPRAE capsules (NC) were tested in SIF after storage. Capsules contained non-dried CA and SB, but no enteric covering. Data were normalized to NC capsules in SIF. Capsules were stored at 15–19% RH at room temperature and pressure. Statistical significance is shown for comparisons of time to burst just while in SIF (*) and total time until burst (&). Welch’s *t*-test determined statistical significance (ns: *p* > 0.05, * *p* < 0.05, && *p* < 0.01, *** *p* < 0.001, *n* = 5–10 replicates per group).

After fabrication and storage in this way, non-covered capsules placed in SIF did not have any visible holes or cracks in the gelatin, and effervescent excipients did not look wet or clumpy. All the capsules burst at the delivery orifice, and burst pressure and pressurization rates were not significantly different after 3 days compared to freshly made capsules (Welch’s *t*-test, *p* > 0.05; Figure 9A). There was also a lag time before pressure release for the stored capsules of 8.48 ± 1.37 min. This indicated that there was initially insufficient water in the capsule for the effervescence reaction, such that it took time for water from SIF to cross the capsule wall and come in contact with the excipients. Total time to burst was not significantly different (Welch’s *t*-test, *p* = 0.0901; Figure 9A) between fresh and stored capsules.

Karl Fischer titration showed that the excipients did not gain a significant amount of water during storage (Welch’s *t*-test, *p* > 0.05; Figure 9B), indicating improved stability over non-dried excipients without controlled-humidity fabrication and storage (Figure 5E). Gelatin in the capsule wall significantly lost water during storage (Welch’s *t*-test, *p* = 0.0167; Figure 9B), which could be due to water loss from the gelatin to the surrounding low-humidity air (15–19% RH), which was less humid than the recommended hard gelatin capsule storage condition (35–65% RH) [18,19,31,32].

We next tested enteric-covered capsules with Tecoflex, dried CA, and controlled humidity after storage and placement in SGF, followed by SIF. Burst pressure, pressurization rate, and time to burst were all similar to before storage (Welch’s *t*-test, *p* > 0.05; Figure 9C), and all capsules burst at the delivery orifice only after being transferred to SIF after 30 min in SGF. All but one sample (75%) had essentially no lag time before pressure increase. Altogether, these data indicate that enteric covering did not affect capsule stability performance.

As a control experiment, capsules were stored with non-dried CA but prepared in the controlled humidity glove box to determine the sole effect of controlled humidity on capsule storage stability (Figure 9D). After storage, these control capsules had similar burst pressure (Welch’s *t*-test, *p* > 0.05), but significantly lower pressurization rate (Welch’s *t*-test, *p* < 0.001) and longer time to burst (Welch’s *t*-test, *p* < 0.01). All capsules burst at the delivery orifice and did not exhibit a lag time after storage. The lower pressurization rate and longer time to burst show that these control capsules were not fully stable, indicating that both drying CA and using a controlled humidity environment are necessary for maintaining capsule performance after storage.

### 3.7. Storage Stability up to 30 Days

**Figure 10:**
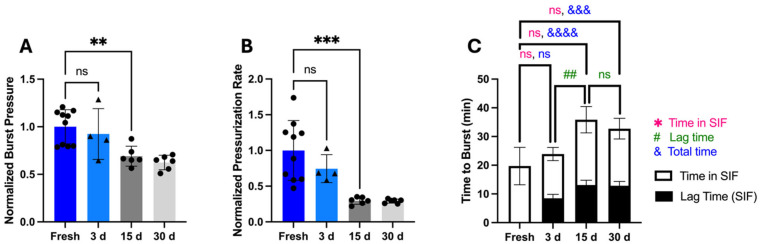
The effect of storage for up to 30 days at reduced humidity on bursting behavior of OSPRAE capsules in SIF. Non-covered OSPRAE capsules containing Tecoflex (NC/Teco) were tested in SIF after storage for 15 or 30 days. Capsules contained dried CA and SB, but no enteric covering. Capsule burst pressure (**A**), pressurization rate (**B**), and time to burst (**C**) were measured. Data were normalized to NC/Teco capsules in SIF. Data for 3 days is the same data in Figure 9A. Statistical significance is shown for comparisons of time to burst in SIF (*), lag time until pressure rise (#), and total time until burst (&). One-way ANOVA was used to determine significance (ns: *p* > 0.05, ## or ** *p* < 0.01, &&& or *** *p* < 0.001, &&&& *p* < 0.0001, *n* = 4–10 replicates per group).

To assess storage stability beyond 3 days, we measured OSPRAE capsule bursting behavior for up to 30 days. After extended storage, capsules burst through the delivery orifice and did not have visible holes in the gelatin even after storage of up to 30 days. However, beyond 3 days of storage, burst pressure (one-way ANOVA, *p* < 0.001; Figure 10A) and pressurization rate (one-way ANOVA, *p* < 0.0001; Figure 10B) were significantly lower, indicating a loss of capsule stability. The time to burst significantly increased (one-way ANOVA, *p* < 0.0001; Figure 10C), and lag time for 15- and 30-day storage was significantly longer than for 3 days (one-way ANOVA, *p* = 0.0008; Figure 10C).

The pressurization rate was calculated as the average over the full incubation time in SIF (i.e., including the lag time, during which there was very little pressure increase). If we subtract the initial lag phase, then the time in SIF until capsule burst did not differ between fresh and stored capsules (one-way ANOVA, *p* > 0.05), and the pressurization rate after the lag time also did not depend on storage time (one-way ANOVA, *p* > 0.05). This indicates that capsule storage primarily affected capsules by delaying the onset of pressure rise rather than slowing pressurization rate.

The increased time until burst time after extended storage, prolonged exposure of the gelatin delivery orifice in SF. This may have reduced burst pressure due to increased gelatin degradation by SIF [14], causing the capsule to burst at a lower pressure. Altogether, we hypothesize that the prolonged incubation time in SIF due to an increased lag time led to a weakening of the delivery orifice, leading to a lower burst pressure after 15 days or longer.

### 3.8. Other Methods to Improve Shelf-Life Stability

Other approaches besides drying CA and using a controlled-humidity chamber to reduce effervescence reactivity during storage were also investigated. Instead of using a controlled-humidity chamber for capsule assembly, desiccant was added to the capsule storage container to reduce water content of the surrounding air during storage. However, 17% of capsules failed after 24 h storage, despite the presence of desiccant (Supplementary Figure S6A). By visual observation, capsules also appeared more brittle after storage. The capsules, which failed to burst at various locations other than the delivery orifice, such as at holes or cracks in the gelatin capsule wall that appeared after storage.

Since desiccant was not sufficient for preventing effervescence pre-reaction, capsules were also stored at a lower temperature (at 4 °C or with dry ice) to reduce reactivity of the excipients. However, cold storage resulted in brittle gelatin that had high failure rates (≥20%) and burst at locations other than the delivery orifice (Supplementary Figure S6A). Since hard gelatin capsules are recommended to be stored at 15–25 °C and 35–65% RH, the addition of desiccant or lower temperature likely compromised the mechanical integrity of the gelatin capsule walls [18,19,31,32].

Using a less hygroscopic acid source instead of CA was also tested, such as fumaric acid [21], but the pressurization rate was too slow (Supplementary Figure S6B). Extrapolating from data collected for almost 1 h, reaching a desired burst pressure of >100 kPa would have taken more than 5 h. Performing wet or dry granulation of the effervescent excipients (commonly used for effervescent tablets [21]) was also briefly tested, but we found that the pressure increase was also too slow after these processing steps. Effervescent excipients were also coated in a thin layer of dissolvable polymer (0.13% *w/w* PVP in ethanol) that could dissolve off in water (e.g., in SIF) but acted as a barrier between the SB and CA during storage. However, pressure increase was very slow (Supplementary Figure S6C), probably because not enough water entered the capsule from SIF to sufficiently dissolve the PVP, so this method was not pursued further.

Instead of a dissolution-triggered barrier between the SB and CA, a thermally-triggered barrier was also made using lipids (Witepsol S55) that were solid at room temperature and only melted above 33 °C [33]. Although the lipid formulation melted when the capsule was placed in SIF at 37 °C, the melted lipid appeared to still block the SB and CA from having sufficient contact to generate a noticeable pressure increase (Supplementary Figure S7).

### 3.9. Relationship Between Burst Pressure and Time to Burst

**Figure 11:**
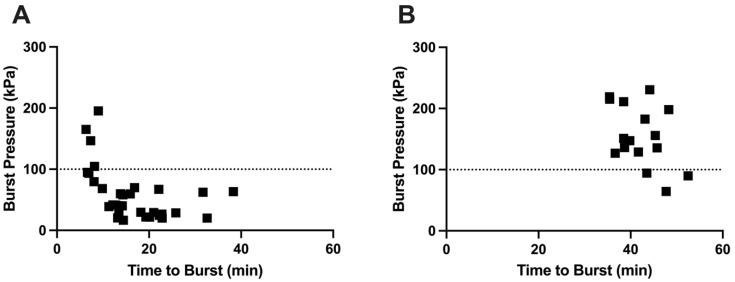
Relationship between burst pressure and time to burst of OSPRAE capsules in SIF. (**A**) Freshly made capsules without enteric covering (NC) tested in SIF. Data from Figure 5 and Figure 6. (**B**) Freshly made capsules with 13 mg enteric covering (EC) tested in SGF for 30 min and then transferred to SIF. Data from Figure 2, Figure 6 and Figure 7. Data include NC capsules with or without Tecoflex, and capsules containing either CA or dried CA and SB. A dashed line is shown at 100 kPa, which is the minimum pressure that was previously shown to effectively penetrate intestinal mucosa [14].

We hypothesized that the highest burst pressures are associated with a short time to burst, while a longer time to burst leads to lower burst pressure. This is because longer burst time is associated with a longer time for the gelatin in the delivery orifice to weaken (and therefore burst at a lower pressure) due to water absorption and enzymatic and thermal degradation [14]. Using capsules without an enteric covering, we observed this relationship, with short burst times associated with high burst pressures and longer burst times leading to lower burst pressure (Figure 11A). In contrast, there was little correlation between burst pressure and time to burst for enteric-covered capsules (Figure 11B), probably due to physical protection and mechanical reinforcement of the delivery orifice by the coating, enabling the delivery orifice to remain strong during long burst times.

## 4. Discussion

OSPRAE capsules offer the promise of administering biologics, as well as other drugs that require injection, via the oral route. These self-pressurized capsules can deliver drugs with higher bioavailability by using high velocity ejection in the small intestine to penetrate across mucosal barriers. OSPRAE is made out of conventional pharmaceutical excipients that can enable low-cost, large-scale manufacturing and do not require any moving parts or mechanical or electrical components.

Two limitations of the original OSPRAE capsule design are an inability to selectively deliver the drug in the intestine and not in the stomach, and the need to use capsules immediately after fabrication due to instability of the effervescent excipients. In this study, we addressed the first limitation by demonstrating that adding enteric covering to OSPRAE capsules can prevent premature drug delivery in SGF (representing the stomach) and allow for delivery selectively in the SIF (representing the small intestine).

We observed that enteric covering provided a mechanical barrier over the capsule delivery orifice that prevented it from bursting while in the low pH environment of SGF. Enteric-covered capsules tested in SGF and then transferred to SIF also had higher burst pressures than capsules without enteric covering in SIF. Since the time until delivery was prolonged while the enteric-covered capsules were in SGF, their internal pressure was able to build up before releasing in the SIF. Adding more enteric covering required more time for the enteric covering to dissolve in SIF, which led to higher burst pressures.

The effect of enteric covering on capsule burst behavior has important translational implications. First, it enables drug delivery to target the small intestine by adapting a well-established capsule-coating technology (i.e., enteric coating). In addition, by changing the mass of enteric covering on the capsule, the time to burst, and thereby the burst pressure, can be modified. A challenge of this approach, however, is that gastric emptying times in humans can range between 0.5–10+ h due to interindividual variability, as well as other factors like fasting, fluid intake, age, gender, disease state, or caloric content of food [34,35,36]. This variable residence time in the stomach may lead to variable burst pressures, where longer residence time leads to building up more pressure before bursting.

Gastrointestinal contractions can also lead to variability in gastric emptying, mixing of the gastric fluids, pH, and caloric content microenvironment around the capsule, and mechanical forces on the capsules [37], which could all cause variability in OSPRAE pressurization and burst performance. Gastric fluid pH is also variable between patients, especially those on proton pump inhibitors that increase stomach pH. Increased gastric pH could lead to faster enteric coating dissolution or variable water permeability through the coating, leading to variable pressurization rates or times to burst [38]. Future studies will need to address these various sources of variability and their effects on the drug delivery process.

To address the second limitation, we hypothesized that capsule instability during storage could be addressed by reducing water exposure during capsule fabrication and storage to delay effervescence pre-reaction. Our hypothesis also accounted for the risk that reducing humidity by too much during fabrication and storage would lead to gelatin capsule brittleness [19]. Reducing water content of the CA alone did not prevent pre-reaction, but simultaneously reducing water content of the CA and of the air inside/around the capsule allowed for improved stability. Reducing water content of only the air inside/around the capsule (and not the CA) also allowed for a similar capsule burst pressure (but significantly longer time to burst and lower pressurization rate) after 3-day storage (Figure 9D). This indicates that the residual humidity of the air in and around the capsule had a greater influence on determining capsule shelf-life stability than water content of the capsule excipients, but a combination of both drying excipients and air was required for maintaining capsule time to burst and pressurization rate.

In addition, reducing water content of the CA reduced its reactivity, necessitating the use of a Tecoflex coating that allowed for faster CA reactivity, likely due to its higher water uptake than the original lacquer coating. Although the Tecoflex coating had higher water uptake, this only resulted in faster CA reactivity when combined with enteric coating (Figure 6D,F), implying that the enteric coating helped achieve greater water penetration through the gelatin wall to contact the excipients. This suggests that not all the water taken up by the Tecoflex coating permeated through the gelatin wall, and that the addition of enteric coating on the Tecoflex may have aided in increasing water permeation across the gelatin wall through chemical and/or physical interaction. To better understand this process, future work should determine the degree of swelling of each individual coating layer [39], thicknesses and variability of each coating, and mechanisms of gelatin permeability changes due to interactions associated with adding coatings.

Humidity of the air was reduced to 15–19% RH, but was not reduced further, since initial studies showed that storing capsules with desiccant resulted in brittle capsules (Supplementary Figure S6). We also considered capsules made of materials other than gelatin that could withstand becoming brittle in low-humidity environments [40], such as hydroxypropyl methylcellulose (HPMC). However, we did not pursue that approach because HPMC cannot be easily crosslinked [41] like gelatin, so fabricating a delivery orifice with tunable burst pressure properties like the current OSPRAE design would be difficult.

Other approaches to prevent excipient pre-reaction during storage were tested, but not further pursued due to the pressurization rate being too slow. These approaches included replacing CA with fumaric acid, wet or dry granulation of excipients, coating excipients in a thin PVP barrier, or placing a thermally responsive wax barrier between CA and SB. Future studies could further optimize these methods or test if a desired pressurization rate could be achieved using these approaches in an enteric-covered capsule, since capsules would have more time to pressurize.

Use of an enteric covering could also enable formulating with less-reactive excipients, since enteric covering delays time to burst, thereby allowing more time for the capsule to build up pressure. Less-reactive excipients could help to prevent excipient pre-reaction to extend shelf-life stability. Acids that could be less-reactive than CA during storage could include ascorbic acid or fumaric acid, and CO_2_ sources that could be less-reactive than SB could include sodium carbonate [21]. Future studies should test these and other excipients instead of CA and SB in combination with the other stabilizing methods used in this study.

Limitations of the current study include the variability in ambient laboratory conditions that required normalizing burst pressure and pressurization rate data to account for the effects of humidity on capsule burst behavior. Future studies should be performed in a controlled-humidity environment to prevent the need for data normalization to the humidity conditions of the laboratory. While extension of shelf life to 3 days will facilitate certain studies, further improvements in humidity control during manufacturing and storage are needed to stabilize capsules on a time scale of years for clinical translation. Mechanical strength of the capsule walls after storage should also be characterized, since it may affect OSPRAE performance. Additionally, characterization of drug stability in OSPRAE capsules will be needed in future studies addressing the delivery of a drug, and will be especially important for biologics due to their general instability and need for specific formulation development [4]. Future studies should also investigate the use of other materials, such as capsule walls made of a material compatible with desiccated storage, effervescent excipients less susceptible to pre-reaction, and a stronger biocompatible coating instead of lacquer. Another design improvement could include selectively adding enteric covering to only the delivery orifice (rather than the entire capsule body). Since the enteric covering primarily functioned as a mechanical barrier over the delivery orifice, a significant amount of Eudragit solution was wasted by application to the entire capsule body. Although intestinal permeation and bioavailability in rats were demonstrated using OSPRAE capsules previously [14], additional validation and bioavailability studies in animal models, especially ones with GI tracts of comparable size to humans, and eventual human studies are needed too. Furthermore, future scale-up of manufacturing should be considered by adapting existing unit operations commonly used in GMP pharmaceutical manufacturing, such as film coating and encapsulation, to produce OSPRAE capsules. Specifications, acceptance criteria, and in-process checks for coating weight gain should be established to ensure uniform and replicable capsule coating and delivery orifice formation upon scale-up.

## 5. Conclusions

This study addressed two major design improvements on a self-pressurizing hard gelatin capsule (OSPRAE) to enable selective drug delivery in the small intestine and improve stability during storage. First, an enteric covering was added to the capsule delivery orifice and shown to delay drug release while in SGF, but still allow for the capsule to burst when transferred to SIF. Thicker enteric covering allowed for up to 4 times higher burst pressure and longer time to burst when transferred to SIF. The second design improvement to OSPRAE was pre-drying CA, adding Tecoflex coating, and assembling in a controlled-humidity glove box to increase shelf-life stability, while minimizing the risk of gelatin capsule brittleness at very low humidity. Capsules maintained similar performance after 3-day storage, compared to the original OSPRAE design, which often pre-reacted after <1 day. Future studies will be needed to further extend storage time, address possible variability introduced by different gastric pH and emptying times, and further assess OSPRAE performance in vivo. These design improvements will facilitate continued pre-clinical study of OSPRAE capsules along the translational pathway to improved oral delivery of biologics and other hard-to-deliver drugs.

## Data Availability

Data is contained within the article or Supplementary Material.

## Supplementary Materials

The following supporting information can be downloaded at https://www.mdpi.com/article/10.3390/pharmaceutics17091156/s1, Figure S1: Pressure sensor setup for in vitro pressure measurements; Figure S2: Effect of time of year of capsule preparation on bursting behavior of OSPRAE capsules; Figure S3: OSPRAE capsules containing SB (without CA or enteric covering) placed in SGF show no pressure increase; Figure S4: Influence of OSPRAE capsule coating on water uptake; Figure S5: The effect of osmosis on OSPRAE capsule water uptake; Figure S6: Other methods to improve shelf-life stability; Figure S7: Non-covered OSPRAE capsules with meltable barrier between SB and CA.

## References

[1] Bucheit J.D., Pamulapati L.G., Carter N., Malloy K., Dixon D.L., Sisson E.M. (2019). Oral Semaglutide: A Review of the First Oral Glucagon-Like Peptide 1 Receptor Agonist. Diabetes Technol. Ther..

[2] Morita H., Matsumoto K., Saito H. (2022). Biologics for allergic and immunologic diseases. J. Allergy Clin. Immunol..

[3] Maniadakis N., Toth E., Schiff M., Wang X., Nassim M., Szegvari B., Mountian I., Curtis J.R. (2018). A Targeted Literature Review Examining Biologic Therapy Compliance and Persistence in Chronic Inflammatory Diseases to Identify the Associated Unmet Needs, Driving Factors, and Consequences. Adv. Ther..

[4] Brown T.D., Whitehead K.A., Mitragotri S. (2020). Materials for oral delivery of proteins and peptides. Nat. Rev. Mater..

[5] Durán-Lobato M., Niu Z., Alonso M.J. (2020). Oral Delivery of Biologics for Precision Medicine. Adv. Mater..

[6] McGonigle P. (2025). How Biologics Have Changed the Drug Discovery Landscape. Annu. Rev. Pharmacol. Toxicol..

[7] Masloh S., Culot M., Gosselet F., Chevrel A., Scapozza L., Zeisser Labouebe M. (2023). Challenges and Opportunities in the Oral Delivery of Recombinant Biologics. Pharmaceutics.

[8] Buckley S.T., Bækdal T.A., Vegge A., Maarbjerg S.J., Pyke C., Ahnfelt-Rønne J., Madsen K.G., Schéele S.G., Alanentalo T., Kirk R.K. (2018). Transcellular stomach absorption of a derivatized glucagon-like peptide-1 receptor agonist. Sci. Transl. Med..

[9] Overgaard R.V., Navarria A., Ingwersen S.H., Bækdal T.A., Kildemoes R.J. (2021). Clinical pharmacokinetics of oral semaglutide: Analyses of data from clinical pharmacology trials. Clin. Pharmacokinet..

[10] Abramson A., Caffarel-Salvador E., Khang M., Dellal D., Silverstein D., Gao Y., Frederiksen M.R., Vegge A., Hubálek F., Water J.J. (2019). An ingestible self-orienting system for oral delivery of macromolecules. Science.

[11] Abramson A., Caffarel-Salvador E., Soares V., Minahan D., Tian R.Y., Lu X., Dellal D., Gao Y., Kim S., Wainer J. (2019). A luminal unfolding microneedle injector for oral delivery of macromolecules. Nat. Med..

[12] Dhalla A.K., Al-Shamsie Z., Beraki S., Dasari A., Fung L.C., Fusaro L., Garapaty A., Gutierrez B., Gratta D., Hashim M. (2021). A robotic pill for oral delivery of biotherapeutics: Safety, tolerability, and performance in healthy subjects. Drug Deliv. Transl. Res..

[13] Srinivasan S.S., Alshareef A., Hwang A.V., Kang Z., Kuosmanen J., Ishida K., Jenkins J., Liu S., Madani W.A.M., Lennerz J. (2022). RoboCap: Robotic mucus-clearing capsule for enhanced drug delivery in the gastrointestinal tract. Sci. Robot..

[14] Palacios J.I., Wood-Yang A.J., Klavohn N., Friesenhahn N., Raman N., Baker N., Ashby G., Prausnitz M.R. (2025). High-velocity delivery of biologics via the gastrointestinal tract by self-pressurized oral capsules. J. Control. Release.

[15] Patra C.N., Priya R., Swain S., Kumar Jena G., Panigrahi K.C., Ghose D. (2017). Pharmaceutical significance of Eudragit: A review. Futur. J. Pharm. Sci..

[16] Nikam A., Sahoo P.R., Musale S., Pagar R.R., Paiva-Santos A.C., Giram P.S. (2023). A Systematic Overview of Eudragit^®^ Based Copolymer for Smart Healthcare. Pharmaceutics.

[17] Kontny M.J., Mulski C.A. (1989). Gelatin capsule brittleness as a function of relative humidity at room temperature. Int. J. Pharm..

[18] Ciper M., Bodmeier R. (2006). Modified conventional hard gelatin capsules as fast disintegrating dosage form in the oral cavity. Eur. J. Pharm. Biopharm..

[19] Chang R.K., Raghavan K.S., Hussain M.A. (1998). A study on gelatin capsule brittleness: Moisture tranfer between the capsule shell and its content. J. Pharm. Sci..

[20] Fordtran J.S., Morawski S.G., Santa Ana C.A., Rector F.C. (1984). Gas Production After Reaction of Sodium Bicarbonate and Hydrochloric Acid. Gastroenterology.

[21] Bertuzzi G., Parikh D.M. (2010). Handbook of Pharmaceutical Granulation Technology. Handbook of Pharmaceutical Granulation Technology.

[22] Evonik (2024). Eudragit Functional Polymers to Take Control of Your Release Profile. https://healthcare.evonik.com/en/drugdelivery/oral-drug-delivery/oral-excipients/eudragit-portfolio/attachment/149083?rev=fd91102acf8d769a56d0716b20544216.

[23] Pagire S.K., Seaton C.C., Paradkar A. (2020). Improving Stability of Effervescent Products by Co-Crystal Formation: A Novel Application of Crystal Engineered Citric Acid. Cryst. Growth Des..

[24] Wang C., Zolotarskaya O., Ashraf K.M., Wen X., Ohman D.E., Wynne K.J. (2019). Surface Characterization, Antimicrobial Effectiveness, and Human Cell Response for a Biomedical Grade Polyurethane Blended with a Mixed Soft Block PTMO-Quat/PEG Copolyoxetane Polyurethane. ACS Appl. Mater. Interfaces.

[25] Alves P., Coelho J.F.J., Haack J., Rota A., Bruinink A., Gil M.H. (2009). Surface modification and characterization of thermoplastic polyurethane. Eur. Polym. J..

[26] M’Bengue M.-S., Mesnard T., Chai F., Maton M., Gaucher V., Tabary N., García-Fernandez M.-J., Sobocinski J., Martel B., Blanchemain N. (2023). Evaluation of a Medical Grade Thermoplastic Polyurethane for the Manufacture of an Implantable Medical Device: The Impact of FDM 3D-Printing and Gamma Sterilization. Pharmaceutics.

[27] Szycher M., Poirier V. (2021). High Performance Tecoflex Polyurethanes in Biomedical Applications. Advances in biomaterials.

[28] Murphy W., Black J., Hastings G.W. (2016). Handbook of Biomaterial Properties.

[29] Yang M., Zhang Z., Hahn C., Laroche G., King M.W., Guidoin R. (1999). Totally implantable artificial hearts and left ventricular assist devices: Selecting impermeable polycarbonate urethane to manufacture ventricles. J. Biomed. Mater. Res..

[30] Verstraete G., Vandenbussche L., Kasmi S., Nuhn L., Brouckaert D., Van Renterghem J., Grymonpré W., Vanhoorne V., Coenye T., De Geest B.G. (2017). Thermoplastic polyurethane-based intravaginal rings for prophylaxis and treatment of (recurrent) bacterial vaginosis. Int. J. Pharm..

[31] Al-Tabakha M.M., Arida A.I., Fahelelbom K.M., Sadek B., Saeed D.A., Abu Jarad R.A., Jawadi J. (2015). Influence of capsule shell composition on the performance indicators of hypromellose capsule in comparison to hard gelatin capsules. Drug Dev. Ind. Pharm..

[32] LFA Machines Oxford Ltd Empty Hard Gelatin #00 Capsules Safety Data Sheet Technical Specifications Intolerance Data. https://www.lfacapsulefillers.com/media/contentmanager/content/documents/Hard_Gelatin_Capsules_SDS_Specs_and_ID_v.1.1.4.pdf.

[33] Zoubari G., Staufenbiel S., Volz P., Alexiev U., Bodmeier R. (2017). Effect of drug solubility and lipid carrier on drug release from lipid nanoparticles for dermal delivery. Eur. J. Pharm. Biopharm..

[34] Abuhelwa A.Y., Foster D.J., Upton R.N. (2016). A quantitative review and meta-models of the variability and factors affecting oral drug absorption–Part II: Gastrointestinal transit time. AAPS J..

[35] Fix J.A., Cargill R., Engle K. (1993). Controlled gastric emptying. III. Gastric residence time of a nondisintegrating geometric shape in human volunteers. Pharm. Res..

[36] Van Den Abeele J., Rubbens J., Brouwers J., Augustijns P. (2017). The dynamic gastric environment and its impact on drug and formulation behaviour. Eur. J. Pharm. Sci..

[37] Koziolek M., Garbacz G., Neumann M., Weitschies W. (2013). Simulating the Postprandial Stomach: Physiological Considerations for Dissolution and Release Testing. Mol. Pharm..

[38] Mohylyuk V., Yerkhova A., Katynska M., Sirko V., Patel K. (2021). Effect of Elevated pH on the Commercial Enteric-Coated Omeprazole Pellets Resistance: Patent Review and Multisource Generics Comparison. AAPS PharmSciTech.

[39] Amaral Silva D., Davies N.M., Doschak M.R., Al-Gousous J., Bou-Chacra N., Löbenberg R. (2020). Mechanistic understanding of underperforming enteric coated products: Opportunities to add clinical relevance to the dissolution test. J. Control. Release.

[40] Gullapalli R.P., Mazzitelli C.L. (2017). Gelatin and Non-Gelatin Capsule Dosage Forms. J. Pharm. Sci..

[41] Faulhammer E., Kovalcik A., Wahl V., Markl D., Stelzer F., Lawrence S., Khinast J.G., Paudel A. (2016). Multi-methodological investigation of the variability of the microstructure of HPMC hard capsules. Int. J. Pharm..

