# Supplementary material for: Stabilization of Self-Pressurized Gelatin Capsules for Oral Delivery of Biologics
Source: Pharmaceutics. 2025 Sep 3;17(9):1156. doi: 10.3390/pharmaceutics17091156 (PMC12472849; doi:10.3390/pharmaceutics17091156)
Supplement: Supplementary file 1 [file pharmaceutics-17-01156-s001.zip › pharmaceutics-3790828-supplementary.pdf]

SUPPLEMENTARY INFORMATION

Stabilization of self-pressurized gelatin capsules for oral delivery of biologics

Amy J. Wood-Yang[1], Joshua I. Palacios[2], Abishek Sankaranarayanan[1], Mark R. Prausnitz[1,2*]

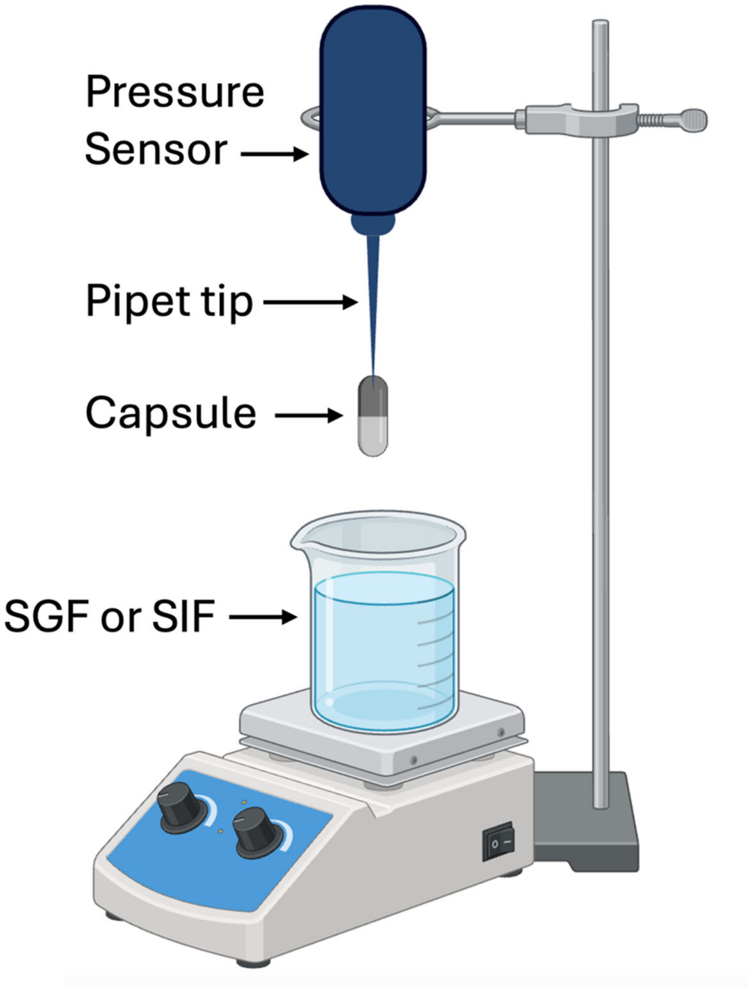

Supplementary Figure S1: Pressure sensor setup for in vitro pressure measurements. Image created with Biorender.com.

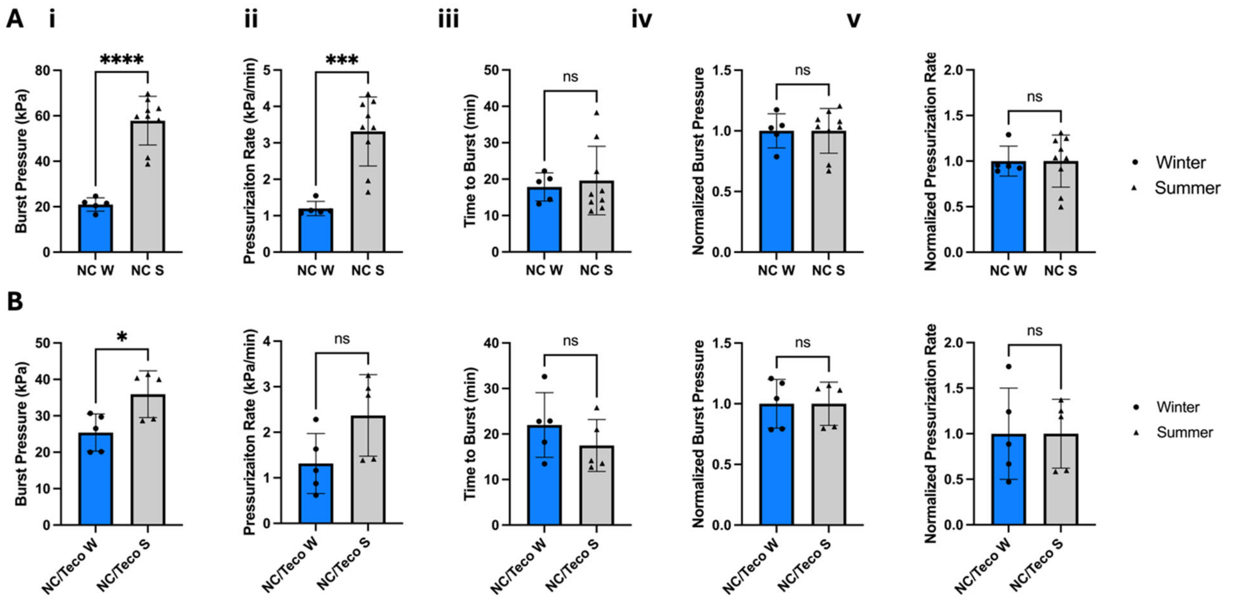

Supplementary Figure S2: Effect of time of year of capsule preparation on bursting behavior of OSPRAE capsules. Winter (W) includes data collected between February and March, and summer (S) is defined as April to September. Capsules contained dried CA and SB. **A)** Capsules with no enteric covering (NC) and **B)** non-enteric-covered capsules with Tecoflex coating (NC/Teco). Welch's t-test determined statistical significance (ns: $p > 0.05$, * $p < 0.05$, *** $p < 0.001$, **** $p < 0.0001$, N = 3 – 9 replicates per group).

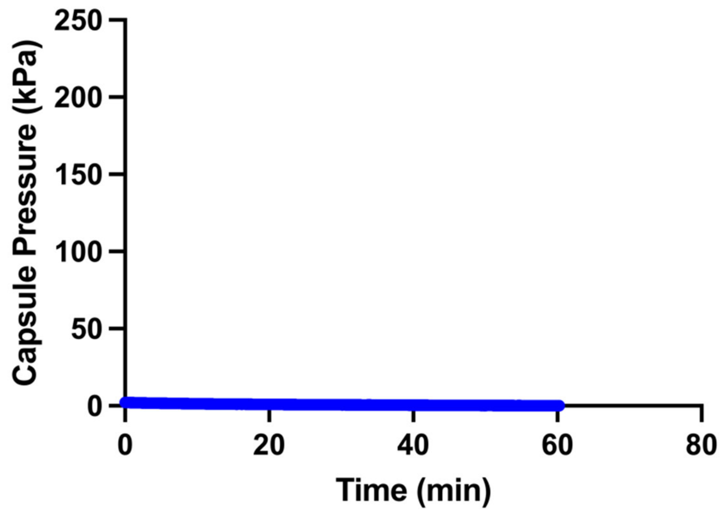

Supplementary Figure S3: OSPRAE capsules containing SB (without CA or enteric covering) placed in SGF show no pressure increase. Capsules started at a slightly elevated pressure due to the residual heat from using UV-curable glue to attach the capsule to the pressure sensor. Data shown are representative of 4 replicates.

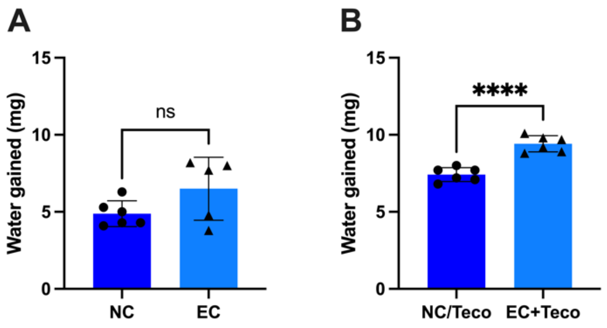

Supplementary Figure 4: Influence of OSPRAE capsule coating on water uptake. Capsules contained model excipients (CA and sodium citrate dihydrate). NC = non-covered, EC = enteric-covered, EC-Teco = enteric covering with Tecoflex. Welch's t-test determined statistical significance (ns: $p > 0.05$, **** $p < 0.0001$, N = 6 replicates per group).

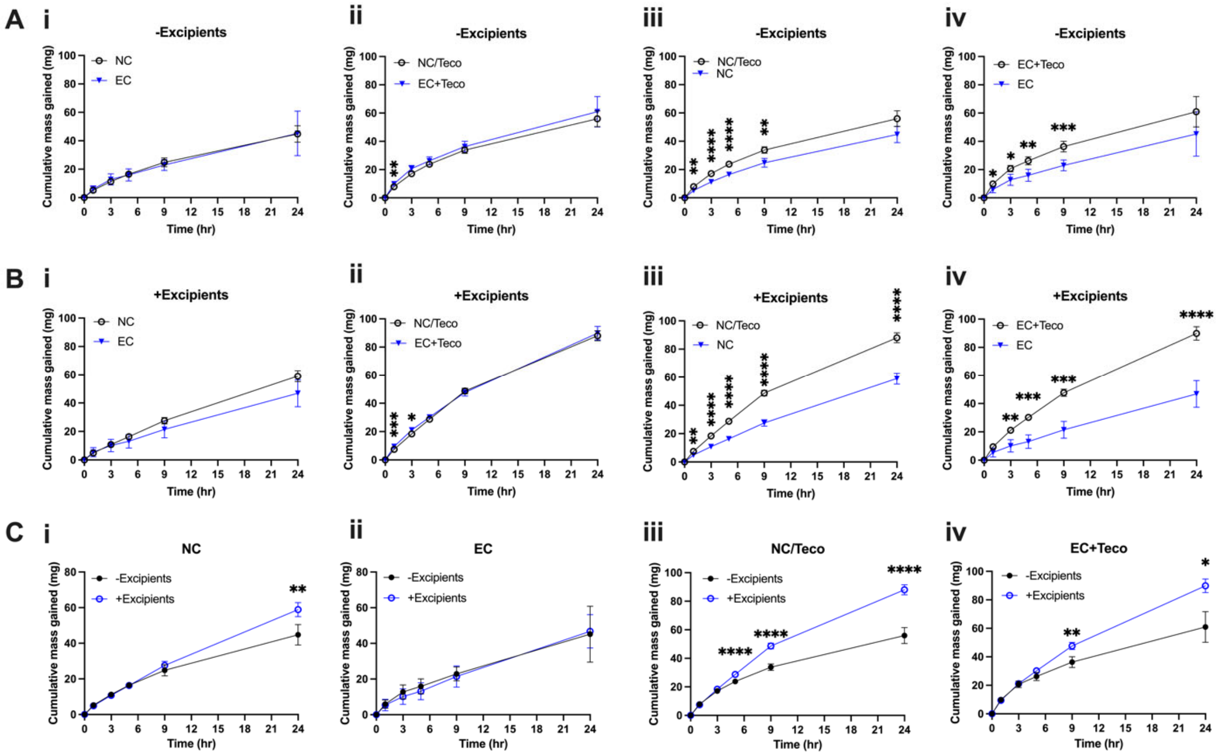

Supplementary Figure S5: The effect of osmosis on OSPRAE capsule water uptake. **A**) Water uptake was measured for capsules without excipients incubated in SIF or SGF. Capsules were either non-covered (NC), enteric-covered (EC), NC with Tecoflex coating (NC/Teco), or EC with Tecoflex (EC/Teco). NC and NC/Teco capsules were incubated in SIF, while EC and EC/Teco capsules were incubated in SGF. **B**) Water uptake was measured for the same types of capsules as in part **A**, but capsules were fabricated containing non-effervescent excipients (CA and sodium citrate dihydrate) to determine water uptake due to osmosis. **C**) The same data from parts **A** and **B** were replotted to compare capsule water uptake with and without the osmotic driving force from excipients in the capsule. Two-way ANOVA determined statistical significance (ns: $p > 0.05$, * $p < 0.05$, ** $p < 0.01$, *** $p < 0.001$, **** $p < 0.0001$, N = 5 – 6 replicates per group). Data points without statistical markers (*) indicates ns.

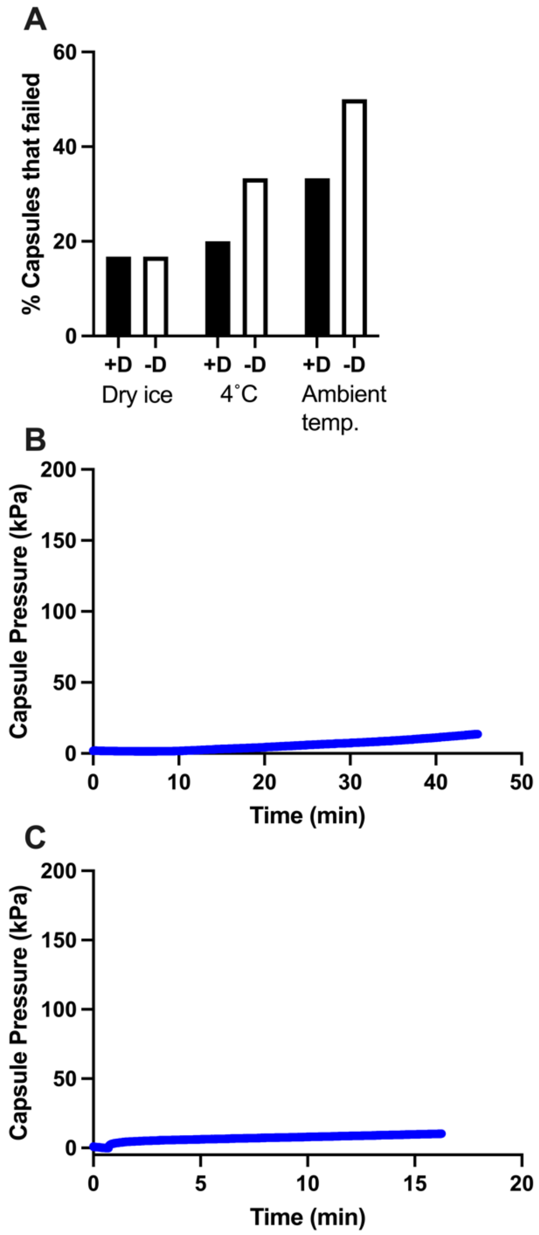

Supplementary Figure S6: Other methods to improve shelf-life stability. **A)** Non-covered capsules (NC) were stored with desiccant (+D) or without desiccant (-D) with dry ice, at 4 ˚C, or at ambient temperature for 24 h, then tested for bursting behavior in SIF. Failure rates determined from 6 replicates. Capsules contained non-dried CA and SB. **B)** NC capsules containing fumaric acid and SB were tested in SIF. Data are representative of 3 separate experiments. **C)** NC capsules containing excipients coated in 0.13% polyvinylpyrrolidone and tested in SIF. Data are representative of 3 separate experiments.

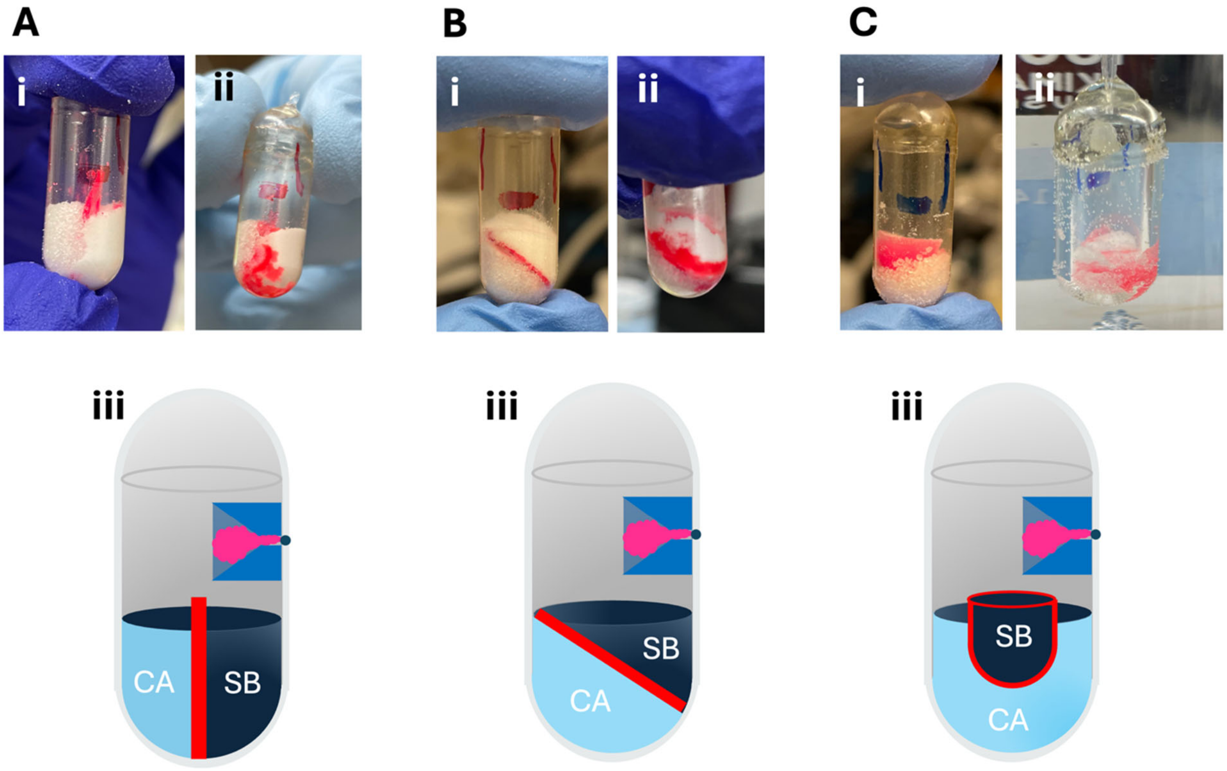

Supplementary Figure S7: Non-covered OSPRAE capsules with meltable barrier between SB and CA. **A)** Vertical, **B)** slanted, or **C)** "capsule-in-capsule" barriers were tested. The barriers were made of a suppository wax that melts at body temperature (Witepsol S55). Representative capsules are shown photographically before (i) and after (ii) testing in SIF, and schematically to show the capsule design (iii). Capsules contained non-dried CA and SB, but no enteric covering.
